# A Review on Auditory Perception for Unmanned Aerial Vehicles

**DOI:** 10.3390/s20247276

**Published:** 2020-12-18

**Authors:** Jose Martinez-Carranza, Caleb Rascon

**Affiliations:** 1 Instituto Nacional de Astrofisica Optica y Electronica (INAOE), Puebla 72840, Mexico; 2 Department of Computer Science, University of Bristol, Bristol BS8 1UB, UK; 3 Instituto de Investigaciones en Matematicas Avanzadas y en Sistemas, Universidad Nacional Autonoma de Mexico, Mexico City 04510, Mexico; caleb.rascon@iimas.unam.mx

**Keywords:** audio, UAV, auditory perception, microphone

## Abstract

Although a significant amount of work has been carried out for visual perception in the context of unmanned aerial vehicles (UAVs), not so much has been done regarding auditory perception. The latter can complement the observation of the environment that surrounds a UAV by providing additional information that can be used to detect, classify, and localize audio sources of interest. Motivated by the usefulness of auditory perception for UAVs, we present a literature review that discusses the audio techniques and microphone configurations reported in the literature. A categorization of techniques is proposed based on the role a UAV plays in the auditory perception (is it the one being perceived or is it the perceiver?), as well as a set of objectives that are more popularly aimed to be accomplished in the current literature (detection, classification, and localization). This literature review aims to provide a concise landscape of the most relevant works on auditory perception in the context of UAVs to date and provides insights into future avenues of research as a guide to those who are beginning to work in this field.

## 1. Introduction

The last years have seen an increase in the number of civilian applications involving unmanned aerial vehicles (UAVs), popularly known as drones. Several applications involve UAVs with on-board cameras aiming at observing the scene, which can be exploited for inspection of critical infrastructure, monitoring, surveillance, and cinema, as well as for live video recording for TV news [1]. Some others include cargo transportation [2], search and rescue [3], and even the use of hundreds of small drones for aerial formations, aiming at replacing fireworks [4].

As mentioned above, visual sensors are amongst the most common sensors to be carried on-board UAVs. This is due to their low cost while providing rich chromatic information of the scene. This has been exploited, for instance, in remote sensing, where drones have become an affordable novel platform that can be easily deployed, thus enabling rapid recording of large volumes of data at much less cost than when operating with larger airborne and spaceborne platforms [5,6].

However, there are scenarios where visual data may not be sufficient to perceive an object of interest. For instance, for robust flight navigation, vision-based processing has been widely used to enable UAVs to detect objects, such as in sense-and-avoid tasks [7,8]. Although high-speed cameras or event cameras [9] could enable detection of very-high-speed moving objects, the main drawback is that the object of interest should be visible in the field of view of the vision sensor. Likewise, if the object is traveling at a very high speed and within the field of view of the sensor, in one frame, it may barely be visible or too small for the vision algorithm to be able to detect it on the image; and in the next frame, the object may appear with a larger size, thus enabling the detection, but with the possibility that the object might be too close by then; hence an avoidance maneuver is prone to failure. In this scenario, an object of interest is desired to be detected with much time ahead, even before it becomes visible to a visual sensor. To address this issue, sense-and-avoid systems are also implemented by relying on laser [10], radar [11,12], and ultrasonic [13]. Nevertheless, audio signal processing is another sensing option that does not depend on detecting the visual shape of the object, provided that the object of interest emits a distinctive sound. This is what we call auditory perception.

Another example where auditory perception has become relevant for UAVs is in search-and-rescue applications. Imagine a situation where a disaster zone is accessible only by UAVs. Smoke in the scene may make visibility difficult. Hence, visual perception may not be enough to detect victims. The UAVs may not be able to get closer to the land; thus, thermal sensing may not get enough data to identify human temperature. Therefore, auditory perception may become useful to complement the searching task by trying to sense people crying out for help.

Given the potential and emerging applications of auditory perception for UAVs, in this document, we aim at reviewing works relevant to this matter. In this context, auditory perception considers the use of one or more microphones to sense the environment, seeking to detect objects of interest via the sound they produce. It is clear that inanimate objects, such as cables, trees, rocks, or walls, are not of interest for this paradigm, but rather those objects whose dynamics are accompanied with sound. Quite interestingly, transitional works between audio signal processing and computer vision have recently been proposed [14,15,16], which employ convolutional neural networks (CNNs)—techniques more commonly used for computer vision. The audio signal is converted into a spectrogram, which can be mapped as a 2D image. It is then passed to a previously trained CNN that classifies whether the sound of an incoming object exists in the spectrogram or not. It is clear that sound detection as a mechanism to detect objects is an area with great potential.

Traditionally, the challenge of sound detection with microphones has been implemented in what we call a land-to-land (LL) scheme. That is, microphones placed at the ground level are used to record the audio signal from objects of interest at the same ground level. This scheme has been effectively used in applications such as service robots [17], internet of things [18], and virtual assistants [19]. In the context of UAVs, it is necessary to define other schemes to encompass different case scenarios. In this review, we propose the use of the following categorization when referring to the three case scenarios where auditory perception is carried out with UAVs:

1. Air-to-Land (AL): The sound originates in the air, and it is sensed by microphones on the land.
2. Land-to-Air (LA): The sound originates on the land, and it is sensed by microphones on-board a UAV.
3. Air-to-Air (AA): The sound originates in the air, and it is sensed by microphones on-board a UAV.

**Figure 1:**
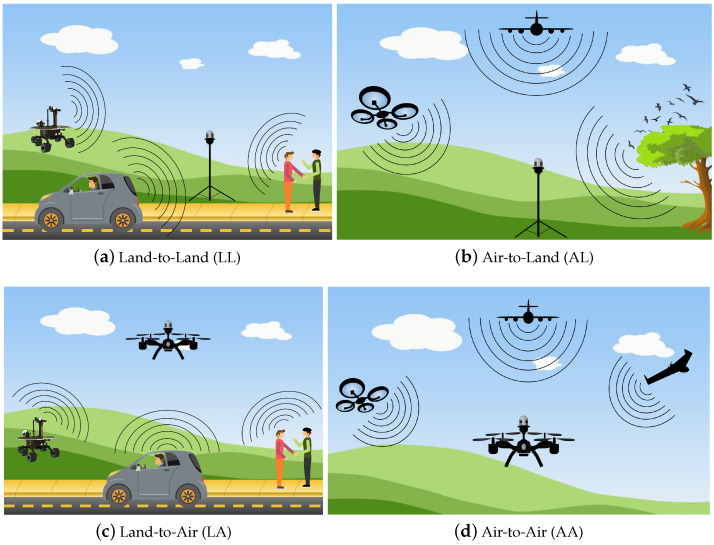
Auditory perception categories defined in this review. The category indicates the origin of the sound and the site where the microphone is placed. Typically, auditory perception has been widely studied for LL. In this review, we focus on auditory perception for unmanned aerial vehicles (UAVs) (**b**–**d**).

In summary, these categories attempt to describe, in a simplified manner, the place of origin of the sound, and (the sound traveling to) the site where the sensor (microphone) perceives the sound. An illustration of these categories is depicted in Figure 1. Note that these categories will be used from now on to categorize the works in the state of the art discussed in this review. By categorizing the works in this manner, several intra-category tendencies emerge, which are discussed further in later sections. It is important to mention that we what we refer to as “land” is inclusive of any type ground point of reference, such as soil, ground, water, etc.

**Figure 2:**
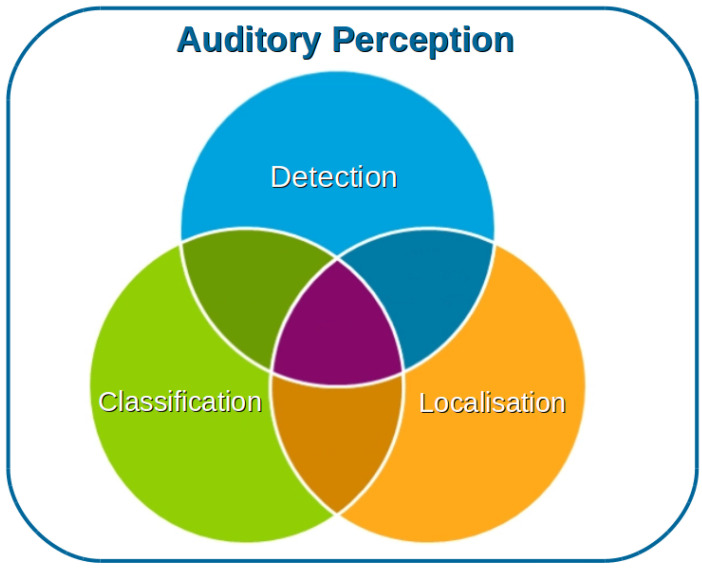
Objectives for which auditory perception is carried out, namely: detection (whether a sound of interest exists or not), classification (what produced the sound), and localization (where the sound is).

In addition to the categories described above, we also aim at distinguishing the objectives of the auditory perception process that are most commonly approached in the context of UAVs. In general terms, these objectives are depicted in Figure 2 and described below:

1. Detection: The signal recorded by the microphones is processed to determine whether a sound of interest is present in the scene.
2. Classification: The signal recorded by the microphones is processed to determine the entity from which the sound originated; for instance, a person, an animal, a UAV, etc.
3. Localization: The signal recorded by the microphones is processed to determine the location of the source. This could be represented by a vector describing the direction of the source with regards to the microphone, with the magnitude representing the distance from the microphone to the source. The angle and a depth value could also be used instead.

These distinctions are useful for inferring the level of difficulty in the research and development of functional systems in the literature. Keep in mind that these objectives are not mutually exclusive. On the contrary, one may think of a complete system as one in which a source is detected, classified, and localized, all in conjunction. However, an initial step to address the auditory perception problem could be that of “detection”, since the motivation would be driven by asserting only whether a particular sound source exists or not in the auditory scene. This is a typical application in search-and-rescue applications, where the objective could be aimed at detecting whether a human is shouting for help regardless of the gender or identity of the human [20,21]. In the same manner, one could be interested in detecting whether a drone flies nearby a facility [22]. However, if we are interested in distinguishing between a quadcopter, a fixed-wing drone, a bird, or a person, then we fall into the “classification” objective [23,24]. In this order, these first two objectives could be accomplished by using one or a few microphones, whereas the last objective (localization) forces us to use several microphones in order to enable direction inference and distance estimation; the reason for this is discussed later. For the sake of brevity, we will refer to “detection” as both objectives of detection and classification, unless otherwise specified.

Having described the categories in which the revised works have been identified (i.e., AL, LA, AA), in addition to the auditory perception objectives (i.e., detection, classification, localization), the rest of this writing discusses in more detail the relevant techniques that such works have used to accomplish their objectives. We begin in Section 2 by providing a brief theoretical background on popular audio techniques and hardware used in auditory perception. Section 3 discusses the number of microphones reported in the works across the years and their relation with the auditory perception categories. Then, in Section 4, Section 5 and Section 6, we provide a more detailed discussion of the revised works by category, aiming at highlighting the key techniques used in these works and their achievements. We continue in Section 7 with a discussion on the opportunities and disadvantages of each auditory perception category, discussing exemplary works and providing insights on what we believe are the next steps for auditory perception with UAVs. For the latter, we present concrete examples of applications, mentioning limitations and open challenges to be considered. We conclude with a summary and final remarks in Section 8. Overall, the goal of this review is to provide a concise landscape of the most relevant works on auditory perception in the context of UAVs to date, hoping that our analysis provides insights into future avenues of research, as well as a guide to those who begin to work in this field.

## 2. Theoretical Background on Audio Techniques and Hardware

We refer to auditory perception as the description of the environment through the analysis of audio signals. This acoustic description of the environment can also be referred to as the auditory scene. For the sake of simplicity, and focusing on application scenarios relevant to UAVs, we can describe the auditory scene by detecting and localizing its sound sources (as described earlier). It is important to emphasize that there are other aspects of the auditory scene that are not included in this point of view (such as separation), but we believe that these aspects are representative of what the state of the art has been acknowledging up until the writing of this work.

### 2.1. Auditory Perception Paradigm

**Figure 3:**
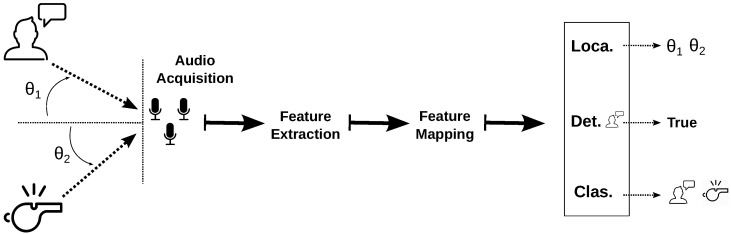
Modular framework paradigm for computational auditory perception: First, audio signals from different sources are acquired with a microphone system; then, audio signals are processed to extract features and and are mapped to a solution space where the transform features can be processed and interpreted to resolve one or more of the objectives in the auditory perception: detection, localization, and/or classification.

Auditory perception in any computational paradigm generally heeds the modular framework shown in Figure 3. It is worth mentioning that the “feature extraction” and “feature mapping” modules are sometimes carried out in conjunction, as in the case of deep-learning-based end-to-end auditory perception.

#### 2.1.1. Audio Acquisition

This module captures the audio from the environment and digitizes it to be used in a computer for analysis. In order to carry out this task, microphones are typically employed (either miniature condensers or Micro-Eletro-Mechanical Systems) and are connected to an audio interface for digitization. Examples of these are the 8SoundsUSB [25] (and its 16SoundsUSB variant) and the Matrix Voice [26].

The digital signal is then transferred to the computer for further processing. Since space is limited in a UAV, the computer can be located off-board, and the digital signal is transmitted to it wirelessly. For real-time applications, it may be necessary to compress the signal to minimize response times, although some compression schemes may hinder localization performance [27]. To this effect, transmission protocols with a small overhead can be considered [24].

#### 2.1.2. Feature Extraction

This module transforms the signals into a domain that is appropriate for the task at hand. From this domain, a set of features is calculated that aims to be representative of the expected type of result. Popular features include:

- Time-frequency (TF) spectrogram: This is a combination of the temporal and frequency domains in one data matrix. The recorded signal is windowed (either by the Hann or Hamming window function), and each window is transformed into the frequency domain via the Fourier transform. Since in most UAV auditory application scenarios, it is not required that the transformed recorded signal is transformed back into the time domain, several methods for data augmentation can be used in the transformation. The number of windows can be incremented by overlapping them, with a 50% overlap being a popular choice. In turn, the size of the windows can be incremented as well, which results in higher frequency resolution. It is important to mention that recently, because of the advent of end-to-end deep-learning-based solutions for auditory perception, it has become common not to calculate any features and to use the transformed signal as the input for the next step of processing. Thus, the TF spectrogram has gained popularity, since it presents not only the spectral shape of the acquired signal, but also how this shape changes through time. In this regard, it provides a two-fold signature of the sound source. In addition, the following features can also be extracted as a type of time-feature matrix if the frequency domain is not representative enough for the task at hand.
- Time difference of arrival between microphones (TDOA): This is the time difference between two captured recordings. There are several ways of calculating it, such as measuring the time difference between the moments of zero-level crossings of the signals [28] or between the onset times calculated from each signal [29]. If the recorded signals are narrowband at frequency *f*, the TDOA is equivalent to the inter-microphone phase difference (IPD), which can be calculated by $\frac{\varphi_{f_{1}}-\varphi_{f_{2}}}{2\pi f}$ [30], where $\varphi_{f_{x}}$ is the phase of recording *x* at frequency *f* in radians. Another popular way of calculating the TDOA is the generalized cross-correlation with phase transform (GCC-PHAT) [31], which maximizes the cross-correlogram between the two recorded signals (CC), computed in the frequency domain as $F\left(CC\right)\left[f\right]=\frac{X_{1}\left[f\right]X_{2}{\left[f\right]}^{*}}{\left|X_{1}\left[f\right]X_{2}{\left[f\right]}^{*}\right|}$, where *X* is the Fourier transform of recorded signal *x*.
- Inter-microphone level difference (ILD): This is the difference spectrum (Δ|X[f]|) between the two short-time-frequency-transformed recorded signals, calculated as $\Delta \left|X\left[f\right]\right|=\left|X_{1}\left[f\right]\right|-\left|X_{2}\left[f\right]\right|$. It can also be calculated in the overtone domain [32], where a frequency $f_{o}$ is an overtone of another *f* when $f_{o}=rf$ (given that r∈[2,3,4,…]). In this domain, the harmonic structures of certain sounds (such as the noise of a UAV motor) are exploited, since the magnitudes between overtones are highly correlated through time.
- Spectral cues: This is a popular term used to refer to a feature set composed of the IPD and the ILD in conjunction.
- Filter bank: The outputs of a set of filters are spaced logarithmically in the frequency domain. The logarithmic spacing is usually set on the Mel-scale [33], which is popularly used to mimic human hearing. A filter bank provides a feature vector with a substantial smaller number of dimensions than the whole frequency spectrum. Additionally, the difference between the filter banks of two recorded signals can also be used as a feature set [34], which has been shown to be robust against noise.
- Mel-frequency cepstral coefficients (MFCCs): An important assumption of a feature vector is that every dimension is non-correlated to every other. Unfortunately, the outputs of a filter bank may be highly correlated to one another. To this effect, the discrete cosine transform is typically applied to the output of a Mel-spaced filter bank to remove correlation between the dimensions of the feature vector, resulting in a set of MFCCs [33]. These features have been frequently used for audio applications and, as seen later in this work, auditory perception in UAVs is no exception.

#### 2.1.3. Feature Mapping

This module provides the estimated result by mapping the features calculated in the last module and maps them onto a solution space. This mapping typically uses a model that takes into account already known characteristics of the auditory scene (such as its propagation model) and/or is trained with data (i.e., a corpus) captured for the task at hand.

This module is the core of most proposed auditory solutions, since it aims to work robustly against unforeseen factors, such as noise, which is a problematic factor in a UAV. There is a considerable amount of types of mapping, which are discussed in the following sections.

### 2.2. Relevant Localization Techniques

Sound source localization estimates the position by sound alone, given a frame of reference. In robotic platforms [17], the polar coordinate system is most commonly used, with its origin at the center of a multi-microphone array, which means that the location of the sound source is given by direction and distance. Depending on the application scenario, the direction can be provided in the azimuth and/or the elevation, corresponding to a one-dimensional and two-dimensional location. A three-dimensional location would then include the distance. Additionally, all of this can be further segmented into single-source and multiple-source localization.

Given the nature of auditory perception in UAVs, the relevant localization techniques are those that carry out two-dimensional single and multiple direction estimation. Distance estimation is usually ignored in UAVs because: (1) It is quite challenging to estimate the distance of a sound source by audio alone even without the enormous amount of ego-noise in place, and (2) it can be estimated by other simpler trigonometric means using the estimation of the UAV’s altitude, which is provided by other sensors.

Furthermore, the elevation direction can be calculated in parallel with the azimuth direction, using the same techniques and a frame of reference shifted 90^o^ in the longitudinal axis. In land-bound robotic applications, the feature most commonly used for one-dimensional single direction estimation is the Time Difference Of Arrival (TDOA) between a pair of microphones and is calculated most popularly by the Generalized Cross-Correlation with PHAse Transform (GCC-PHAT) [31] due to its robustness against moderate reverberation. However, in applications dealing with UAVs, ego-noise is an important issue that GCC-PHAT is known to be sensitive towards; thus, prior noise removal is essential, or other noise-robust techniques are more relevant.

A popular choice, which can also carry out multiple direction estimation, is that of Multiple Signal Classification (MUSIC) [35], which takes advantage of the fact that the eigenvectors that are located in the noisy signal subspace are orthogonal to direction vectors that are located in the source signal subspace. This orthogonality-based model is used to test different direction vectors to find the ones that are highly orthogonal to the “noisy” eigenvectors. Unfortunately, the calculation of these eigenvectors requires the estimation of the covariance matrix at each frequency of interest, which makes it difficult to carry out on-line. To this effect, it has been found [36] that a much faster alternative is to use the Generalized Singular-Value Decomposition (GSVD) instead of the generalized eigenvalue decomposition. This modification is currently an option for sound source localization in the popular robot audition package from the Honda research institute Japan Audition for Robots with Kyoto University (HARK) [37].

Another popular choice is that based on spatial filtering or beamforming, which aims to reduce the energy of another sound source not located in the direction of interest (DOI). The model used by these techniques is the energy in the output of each beamformer that is aimed in each possible direction. Although faster than the MUSIC-based techniques, it does require a high amount of computational power to carry out in an on-line manner. Further enhancements have been made, and a popular choice [38] re-framed the beamform outputs in terms of cross-correlations (which are simpler to calculate) and carried out the search of high-energy outputs in two phases (the second one being a much more refined search in the areas found by the first phase). This technique is currently used for sound source localization in the popular ManyEars robot audition package [25] and has been further enhanced in the Open embeddeD Audition System (ODAS) project [39].

Finally, there has been a recent surge of deep-learning-based sound source localization solutions [40,41,42]. It is worth mentioning that in most of these cases, the short-time Fourier transform was used as the input of the trained models. In all these cases, an extensive training corpus needed to be captured, which may be considered as non-ideal in certain case scenarios. However, at the moment of this writing, these techniques have vastly outperformed the aforementioned ones. Thus, the trade-off between these two issues does need to be considered when deciding upon which to use.

An important factor is implied for all these techniques: They require multiple microphones, since they employ the slight differences between the captured signals to determine the direction of the sound source. Although localization through single-channel data is possible, it requires an important amount of data to train [17], in addition to the data used to make the model robust against ego-noise. Thus, the common usage traditionally employs several microphones, with a rule of thumb of “the more, the better” [17].

### 2.3. Relevant Detection/Classification Techniques

As mentioned before, we refer to “detection” as the process of establishing if a sound source is present in the captured audio signal. This can also be defined as a type of verification task of which, there are a wide variety of methods [43]. Further, “classification” extends this process to establish the type of sound source (human, UAV, noise, etc.) [43]. As also stated before, for the sake of brevity, we will refer to “detection” as both detection and classification.

Both types of processes follow a scheme similar to the localization procedure. A set of features are usually extracted from the audio signal and are decided upon based on how representative they are to the detection task. Popular features for this are the aforementioned Mel-frequency cepstral coefficients, i-vectors [44], and x-vectors [45]. These are then fed to a trained model that presents a distance of the extracted feature set to the expected feature set of a sound source class. Popular models are support vector machines, deep neural networks, and probabilistic linear discriminant analysis, or, if the feature set is robustly representative enough, simply the Euclidean distance or inner product between both the extracted and the expected feature sets can be used.

It is important to mention that, given the nature of the air-to-air case scenario, a UAV can be both the detected and the detector. In the case of it being the detected, the aforementioned schemes can be carried out using on-ground microphones with few to no changes required in already established feature sets and modeling techniques. However, in the case of it being the detector, the significant amount of ego-noise the UAV outputs from its motors considerably reduces the detection performance of traditional workflows [46]. With a substantially large training corpus captured in real-life settings, deep-learning-based methods have shown good detection performances using as their input the signal transformed to the time-frequency domain [15,16].

## 3. On the Number of Microphones and UAV Models

In this section, we present two relevant aspects of the works analyzed in this review. The first one refers to the number of microphones used for recording the audio signal. The second one refers to the UAV models involved in these works. For the former, we aim at discussing the distribution of works over the years in terms of the number of microphones and how this relates to the auditory perception categories and the objectives of the perception. We also discuss the relation between the number of microphones and the UAV models used in the research works. It is interesting to note that, in most of the works, small and micro-UAVs are amongst the most common aerial platforms. Among the latter, quadcopters and hexacopters are a common choice for carrying microphone arrays.

**Figure 4:**
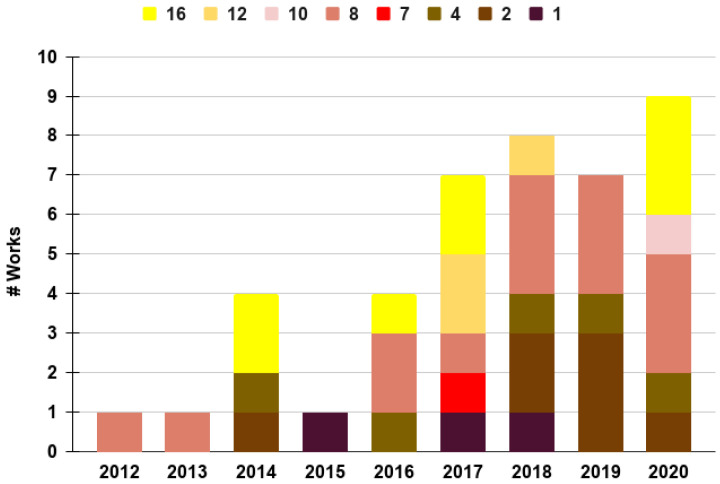
Distribution of works over the years analyzed in this review related to the number of microphones reported. Note that about 25% of the works reported the use of one or two microphones, likely to be associated with a sound detection objective in the auditory perception. In contrast, more complex objectives, such as classification and localization, incentivize the use four or more microphones.

We begin by discussing Figure 4, which shows the distribution of works analyzed in this review across the years distinguished by the number of microphones used to perceive the audio signal of interest. Note that the darker the color, the lower the number of microphones; the lighter the color, the more microphones are used. From this chart, it is interesting to notice that in the last three years (2018–2020), most of the works have used at least two microphones, with a tendency to use a larger number. Additionally, in almost every year, several works have used at least eight microphones or more. We argue that this is due to the interest of the community in addressing the localization problem, since, as will be seen later on, the vast majority of multiple-microphone works aim to localize sound sources.

**Figure 5:**
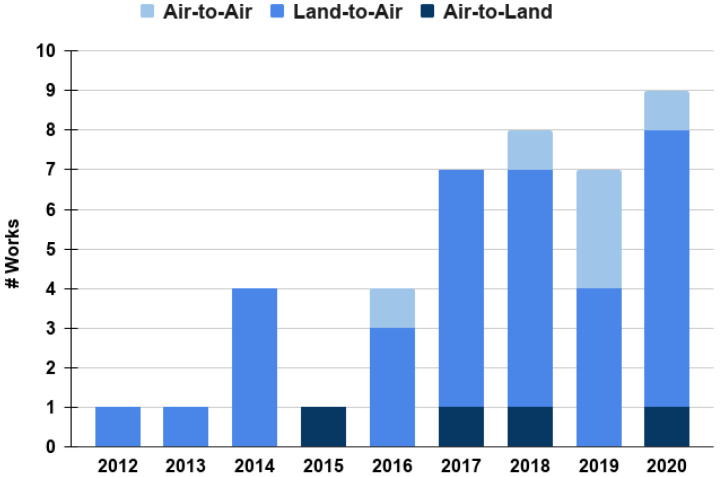
Distribution of works over the years according to the categories of auditory perception as defined in this review: air to land, land to air and air to air. Note that the category with the least number of works is air to land; we have identified that these works are usually related to the detection objective. Air to air also has a few works that have been reported in the last five years (2016–2020).

Table 1 breaks down the number of works analyzed in this review—using from one to sixteen microphones and grouped in one of the three categories corresponding to AL, LA, and A.A. The first thing to notice is that for the AL category, seven works use one microphone, while only one work uses ten microphones. This is due to the fact that (as discussed later) most of these works focus on detecting sound sources originating in the air with a microphone on land (see Figure 1b). Additionally, the majority of works fall into the LA category, where the minimum number of microphones is two, and two-thirds of these works use more than eight microphones. The LA category is mostly associated with the localization task, for which UAVs or aircraft are equipped with an array of microphones, seeking to detect audio sources, but also seeking to provide direction and, wherever possible, the distance of the audio source with regard to the microphones. Among the applications for these works, we find search-and-rescue applications, surveillance and monitoring, and, particularly interestingly, outer-wall inspection of buildings using the drone itself as a reference sound source [47]. Last but not least, only six works are found in the AA category, with three of these works using eight microphones. This is, in fact, the category with the least number of works, but, as has been shown in Figure 5, most of these works have been published in the last three years.

Furthermore, Table 2 indicates the types of UAVs or models (if commercial) used in the research works analyzed in this review, grouped by year and number of microphones. Note that if a work carried out auditory perception in the AL category, then the UAVs used in such works are marked with a * before the UAV type. If the UAV is a custom or hand-made UAV, then we refer to it as “Quad” and “Hexa” as short for Quadcopter and Hexacopter. Regarding the number of rotors, it is worth noticing that the quadcopter is the most common UAV used in these works, even when having to carry microphone arrays of twelve or sixteen microphones. Equally interestingly, works in the AL category have carried out detection of commercial UAVs, which is somewhat expected; as the UAV is seen as an object of study, no special modifications or customizations have to be made. In contrast, for works falling into the LA and AA categories, UAVs have to be adapted to carry out the microphone arrays; hence, custom UAVs may be more convenient.

Overall, there are some interesting tendencies that are worth pointing out at this moment. In Figure 5, we present a distribution of works revised in this review across recent years and differentiated by their category, i.e., AL, LA, and AA. It stands out that the majority of works focus on the LA category. We argue that these have been driven by the increase of search-and-rescue applications involving UAVs. In these applications, the objective is to use UAVs from different types, aiming at detecting audio signals originating on the land where the disaster occurred. Surveillance from the air could also be a potential application; however, it is less common. In contrast, we have found more works falling into the AL category than the AA category. The former could also be related to surveillance and monitoring, where it is desired to sense flying objects near facilities on the land, e.g., birds, UAVs, or aircraft in general. It is important to mention that most of the works in this category are now available commercially “off the shelf” and their methods are not publicly available. Hence, such works may not necessarily be included in Figure 3. The last category, AA, is less common and more recent. We argue that this is a new venue of research that is worthy of being expanded, considering that in the near future, civilian applications will demand the robust and secure integration of UAVs in the air-space. This implies that different types of aircraft and UAVs will navigate in a shared air-space, which will make it necessary to exploit audio signal processing as a complementary measure to sense nearby flying entities. The next sections will discuss in more detail the works falling into the three auditory perception categories mentioned before, but with a closer look at the techniques used for the objective of auditory perception.

## 4. On-Ground Auditory Perception of UAVs (Air to Land)

It was found that the majority of air-to-land applications were primarily employed as acoustic counter-drone systems [82]. In this area, the tasks of detecting and localizing a drone were both carried out. There is a considerable amount of commercial off-the-shelf counter-drone systems; however, few use acoustic sensing to do this: No more than 40 out of 537 known products employ acoustic sensing in conjunction with another modality, while only 10 rely solely on acoustic sensing [83]. Considering that most of these works do not publish their methods, in this section, we aim to provide representative examples of those that are publicly known.

In terms of detection, the work of Jeon et al. [59] carries out this task by exploring several types of models, such as Gaussian mixture models, as well as convolutional and recurrent neural networks. They were trained using augmented recorded data, and obtained F-scores above 80%. Moreover, the work of Park et al. [54] recorded the acoustic frequency profiles of several drones and used a neural network to verify which specific drone was flying nearby. This work also employed radar sensors as part of a multi-modal detection; both radar and acoustic data were integrated through a simple voting system. It is important to state that since, traditionally, a detection task does not require a multi-channel input, these works only employ one microphone.

As for localization, several microphones are typically employed. Such is the case of [78], where the authors employed a 10-microphone array with a beamform-based approach (helped with a Kalman filter) to localize a UAV.

It is important to mention that localization and detection are of interest to be carried out in conjunction in this category. For example, the work of [84] employed a low-cost strategy to locate and detect broadband sources, such as noise-making UAVs. More recently, the work of [85] used a 16- and a 40-microphone array spread over several microphone nodes, estimated the direction of arrival of the UAV, and classified it via its acoustic signature.

It is also important to note that these works obtained a high accuracy of detection and localization (although below a 300 m range), which is laudable considering that the majority of these works use real recorded data to train their models.

Interestingly, there is no clear tendency of technique popularity, since there is a wide variety, from traditional modeling techniques, like Gaussian mixture models, to more recently developed techniques, like recurrent neural networks.

The types of features used also varied: Mel-frequency cepstral coefficients, Fourier-transformed signals, time-frequency spectrograms, and beamformer-based approaches were all used. As for the latter, it is important to mention that typical sound source localization methods [17] are appropriate for acoustic counter-drone systems, and we suspect that they may be employed frequently in the aforementioned commercial off-the-shelf counter drone systems [83].

## 5. Auditory Perception from a UAV (Land to Air)

It was found that the majority of land-to-air works focus primarily on locating a land-bounded sound source from a UAV. Table 3 presents an overview of such works.

In terms of land-bounded sound source detection from a UAV, there are only a few works that carry it out. The work of [57] divides the work into two different deep networks (to reduce computational costs): one that separates the signals and another that classifies them (fed by MFCC features). The authors extended their work to use partially annotated data [56]. Similarly, there are also a few works that carry out localization and detection in tandem. The work of [81] uses a variation of MUSIC to localize sound sources, the features of which are fed to a support vector machine (SVM) for classification. The authors extended their work to separate each sound source before carrying out classification [79].

Additionally, the recent LA work of [76] does not aim to localize either, but to enhance the signal being captured from the ground via a technique based on deep learning. This work can have important implications for further localization or detection from a UAV.

Returning to the topic of localization, the vast majority of the works in Table 3 only estimate the azimuth and the elevation angle of the sound source (or can be trivially extended to estimate both). Although the distance of the sound source can be easily calculated with the elevation angle of the sound source and the altitude of the UAV, this approach is very sensitive to estimation errors in either. Thus, the work of [75] estimates the sound source distance by employing several UAVs for triangulation and redundancy, while the work of [21] carries out several estimations while the UAV is moving for the same purposes.

An important number of works in Table 3 only localize one sound source at a time, which is to be expected, since some of the employed techniques assume only one active source at a time or assume that the UAV itself is a sound source in the environment (which is then actively ignored or cancelled out). However, there are exceptions to this. The work of [75] employed several UAVs to locate multiple sound sources. The work of [80] used the global nearest neighbor to cluster Direction of Arrival (DOA) estimated through time using the sound source features; then, the authors used a variation of MUSIC to estimate multiples DOAs at a time [79,81].

Additionally, there is almost a 50/50 split in terms of using real-life recordings or simulated data to test the localization techniques, which implies that there is a need to standardize evaluation methodologies. In fact, as can be seen in Table 3, there is not even a standard performance metric used by all the works, which makes them difficult to compare.

In terms of techniques used, it seems that MUSIC (and several variations of it) is one of the more popular techniques. This is expected, since it has proven to be quite robust against noise (which is quite prevalent in UAV scenarios). In fact, most of the works that employ this technique do not use any other type of additional noise removal. To be fair, however, most of the works presented in Table 3 that use MUSIC for localization are from (or directly involved with) the HARK group, led by Okuno and Nakadai, who are quite prolific in the area of drone audition. The techniques of the rest of the works, although quite varied, uphold a similar auditory paradigm to the one presented in Figure 3, since they tend to:

1. (Optionally) carry out noise removal from input signals, such as Time-Frequency (TF) filtering, Improved Minima Controlled Recursive Averaging (IMCRA) for noise estimation, Wiener filtering, diagonal unloading (during the beamforming process), etc.
2. Extract features from which to estimate angles, such as inter-microphone time difference of arrival, directional energy response (via beamforming or Signal-to-Noise Ratio (SNR) measurements), spatial likelihood, etc.
3. (Optionally) employ a tracking algorithm for mobile sources, such as Kalman filtering, particle filtering, global nearest neighbor, etc.

Although some works do not uphold this exact protocol, we believe that it is representative of the overall approach that has been employed up until this writing. In fact, one could argue that even the recent work of [74] (which is the only one in Table 3 that uses a deep-learning-based approach for localization) upholds this protocol as closely as the oldest non-MUSIC work reported here [50], albeit with much better reported performance. This observation is consistent with what has been reported in recent search-and-rescue drone competitions, such as the IEEE Signal Processing Cup 2019 Student Competition [73].

It is important to note that in a considerable number of non-MUSIC approaches reported in Table 3, the UAV’s ego-noise is removed by exploiting the harmonic structure of its rotors’ acoustic spectral signature. This seems to be effective, since techniques that are sensitive to noise (such as GCC-PHAT used in [60] and cosine distance between signals in [70]) provide a moderate to good performance after noise removal.

**Figure 6:**
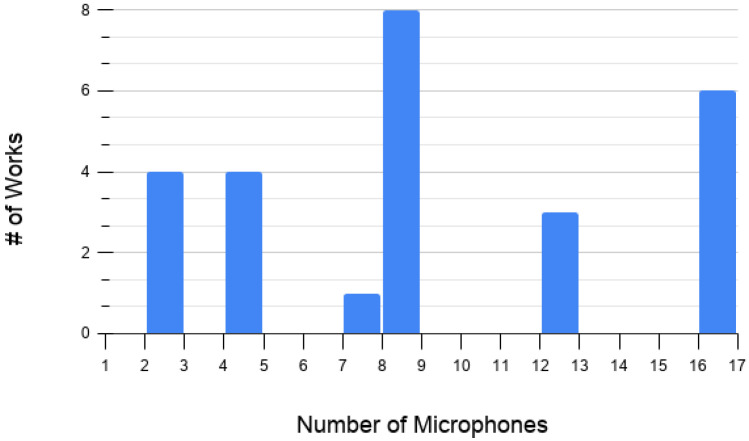
Number of works with respect to the number of microphones that have reported localization techniques and falling into the land-to-air category. Note that the largest number of works report the use of eight microphones, followed by sixteen microphones.

In terms of the number of microphones employed, as can be seen in Figure 6, the most used amount of microphones for locating a sound source from a UAV is eight. However, 16 microphones are also popular. This is expected, since many of the techniques that are used in Table 3 (MUSIC variations, spatial features, and beamforming) benefit greatly from a considerable number of microphones.

## 6. Auditory Perception of a UAV from Another UAV (Air to Air)

Very few works were found where a UAV localized/detected another UAV through audio. In terms of localization, the work of [65] exploited the harmonic nature of the noise generated by a propeller-driven UAV through a constant false alarm rate (CFAR)-enhanced method. Their subsequent work [69] employed a grid-search approach that also exploited the UAV’s noise harmonic nature, but through a spatial beamformer to improve DOA accuracy this time. A grid-search approach was also employed in [55], but in this case, a coherence metric was calculated based on the TDOA between microphone pairs.

**Figure 7:**
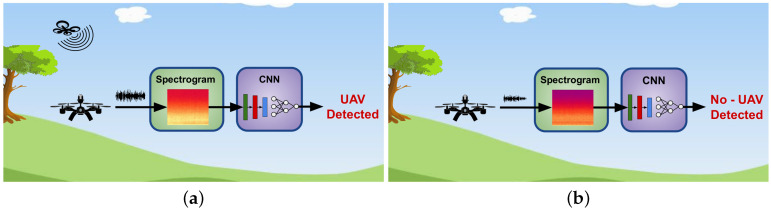
Illustrative example of convolutional neural network (CNN)-based auditory perception for the air-to-air category. In this example, the objective is to carry out UAV detection, that is, whether a UAV is in the scene or not. The audio signal acquisition is performed by another UAV. The audio signal is converted into a spectrogram image, which is passed to a pre-trained CNN model. This network has been trained to output two classes: (**a**) UAV Detected; (**b**) No UAV Detected.

In terms of detection, the work of [15,16] (both works from the same group) used an eight-microphone array mounted on a quadcopter. An Inception v.3 convolutional neural network was trained with TF spectrograms and consistently obtained an accuracy above 90%. These works reported experiments for off-line and on-line processing. Figure 7 illustrates this CNN-based auditory perception for the AA category, focusing on the detection objective. In the depiction, a pre-trained CNN model outputs two labels: “UAV Detected” or “No UAV Detected”, depending on whether a UAV is around in the scene or not. Note that the audio signal is transformed into a spectrogram image, a useful input format for CNNs.

In a similar approach, Ref. [14] used an acoustic camera with eight microphones for data recording. This work was aimed at detecting airplanes near a UAV. However, given the complexity of testing with real planes flying near UAVs, the authors decided to assemble a dataset by recording separate audio sources from airplanes and the UAV propeller noise, which were fused together to emulate an AA scenario. The authors discussed the importance of data labeling and the effect of ego-noise, which is recommended to be reduced as much as possible.

Overall, it is worth noticing that no work was found that carried out both localization and detection of a UAV from another. As can be deduced, there are only a few works that localized or detected a UAV from another UAV. This may be explained by the challenge of having to deal with both the acoustic dynamism of the “perceiving” UAV and the scenario variability of the “perceived” UAV. That is to say, we argue that the AA case scenario is challenging due to three main factors:

1. Limited payload can restrict the hardware to be mounted onboard, including microphones and processing units.
2. The considerable amount of ego-noise forces researchers to either:
  (a) Place the microphones as far as possible from propellers or motors, or
  (b) Identify and characterize it to either:
    i. Remove it by pre-processing means, or
    ii. Consider it as part of the employed technique.
3. Aerial audio sources are highly mobile.

However, it is of interest to take on the challenge of AA auditory perception, since some security applications may benefit from it [15].

## 7. Discussion: Next Steps of Audio with UAVs

This section aims at discussing potential applications of auditory perception for UAVs, their limitations, and open challenges that we deem to be valuable avenues of research.

### 7.1. Disadvantages of Each Category

Before continuing, we believe it is important to establish that carrying out auditory perception in each of the method categories (air to land, land to air, and air to air) bears disadvantages that warrant discussion.

For the air-to-land category, the static nature of the acquisition hardware implies that only a certain space is monitored for UAVs and, since acoustic signals lose energy through the air quite rapidly, these methods have a relatively small sensing range [83]. A way to work around this is by using a large number of microphones spread over a larger area and connected through wireless protocols, as carried out in [85]. However, the cost of doing this is considerable and may even be impractical in some scenarios.

For the land-to-air category, unless the ego-noise of the UAV is dealt with at the physical/hardware level, this type of method needs to be robust against signals with a very small SNR. Pre-filtering techniques can be used to work around this issue, and have shown good results, but they may impose their own set of issues, such as frequency coloring, signal dealignment, and artifact insertion. Another way to do this is to make the noise-robustness part of the method itself (as with MUSIC and its variations), but they have high computational requirements, which make them inadequate to be run on the UAV’s on-board computer, resulting in needing an offsite computer to run. This, in turn, results in shortening the space where the UAV may be able to roam (so as to continue to have wireless reception), which nullifies the main benefit of auditory perception over a UAV.

For the air-to-air category, the issues of the other two categories are mixed into one. The sensing range is small, and, although the UAV may be able to move towards the detected UAV to bridge this gap, the method needs to be both robust against ego-noise and not require as much computational power. In addition, the ego-noise robustness needs to be able to distinguish between the noise that is coming from the sensing UAV (which is to be ignored) and that of the sensed UAV (which is to be detected). All of this in conjunction results in a very challenging case scenario that, in a way, explains the small number of currently published works that aim to solve it.

### 7.2. Representative Examples

In earlier sections, details of each category were discussed in a general sense. However, there are details that are discussed more appropriately when considering representative examples of each category.

For the air-to-land category, there is a vast number of examples that are commercially available off the shelf, but few openly publish their methods. Of the ones that are available, few carry out localization and detection in conjunction. For localization, the work of [78] uses a delay-and-sum (DAS) beamformer applied on a 3D grid to search for high-energy directions to propose a direction candidate. Then, a Kalman filter is applied to smooth the sound source trajectory. For detection, the work of [59] carried out an exploration of three different types of models: Gaussian mixture models, recurrent neural networks, and convolutional neural networks. Mel-frequency cepstral coefficients (MFCCs) were used as training features. It was found that, even though the recurrent neural network outperformed the other two models, Gaussian mixture models required less computational power. It was also found that no noticeable characteristics were able to be extracted from recordings of drones that were farther than 150 m apart; this is consistent with the common knowledge that acoustic signals are not appropriate for large distances [82,83].

For the land-to-air category, the work of [79] is representative of this category’s efforts: carrying out the localization and detection of multiple land-bound sound sources. The sound sources are localized using a variation of MUSIC (called GSVD-MUSIC), the results are associated into clusters through time, and a Kalman filter is applied to each associated cluster to remove uncertainties caused by the UAV ego-noise. Next, using the localization of each sound source, the audio data from each sound source are separated into different channels using a beamform-based approach called geometric high-order decorrelation-based source separation (GHDSS). Mel-separated filter banks are then extracted from each separated channel and fed as training features to a support vector machine (SVM) for sound source detection and classification. The system was evaluated with simulated data and validated with field tests with good results. As can be seen, the paradigm shown in Figure 3 is paralleled in this work, with feature extraction including source separation. The authors state that the decision of the method to use for sound source localization and separation was based on the group’s prior experience, specifically that of the robot audition software library HARK. This implies that even though there is an important overlap between auditory perception in service robots and UAVs, there is a need to complement them to make them robust against ego-noise to make them viable for UAVs.

For the air-to-air category, a situation arises that is similar to the air-to-land category, in which there are not any works that carry out localization and detection in conjunction. As for localization, the work of [69] uses a grid-search approach. The time-difference of arrival (TDOA) is calculated between microphone pairs, and then a coherence metric is measured between these TDOAs to make the localization method robust against ego-noise. As for detection, in the work of [16], the overlap between the ego-noise of the sensing UAV and the noise of the sensed UAV was worked around by feeding time-frequency spectrograms to an Inception v.3 neural network. Both works obtained good results.

### 7.3. Applications

In terms of application scenarios, the most popularly observed is that of search and rescue, where drones are used to localize and sometimes detect human victims. Another is that of air-space policing, in which audio is used to establish if an unauthorized UAV is flying over restricted air-space. However, we believe that there are other application scenarios that, as far as we know, have not been exploited:

- Bioacoustics: Obtaining a census of individuals of an animal species in an ecosystem is of interest for ecological purposes. Unfortunately, carrying out a census usually involves a considerable amount of preparation (both in human and logistical resources). Even if it is done through audio processing alone, the placement and recovery of audio acquisition hardware is labor-intensive, which results in data with a very low time resolution. Doing so by employing automatized drones would simplify data acquisition, and may even improve time resolution if the audio analysis is also carried out on-board.
- Urban security: Vision-based surveillance can only go so far in detecting and localizing crime-related activity, since the visual range is limited and can be relatively costly to expand. Audio-based techniques can provide an initial estimate of the direction of where a crime-related sound source is located, which can then be used to point the on-board camera.
- Collision avoidance: As mentioned before, because of UAVs’ high mobility, vision-based techniques may be too slow to provide information when avoid colliding with another UAV. On the other hand, audio-based techniques can be light and quick enough to provide an initial basis from which to move to avoid a collision, which can complement automatic navigation. It is noteworthy that the work of [69] does carry this out, but it is a rare exception, and we believe it would be beneficial if it was more prevalent.
- Air-space policing through other UAVs: Although air-space policing through auditory perception is now quite common [82,83], it has been shown in this literature that the vast majority of works that do this belong to the air-to-land category. Furthermore, Figure 5 shows that the AL works have not been as prolific as those of the land-to-air category. To take advantage of the fact that UAVs are already able to locate land-bound sound sources, we believe that this application scenario could benefit if more works are focused on locating other UAVs. The works belonging in the AA category could be focused on this issue relatively easy.
- UAV swarm control: Moreover, having mentioned air-space policing through other UAVs as a possibility, a reasonable next step is to use that information to control a swarm of UAVs in a simplified manner. This means that if a UAV is able to locate other UAVs around it, the swarm of UAVs can be programmed as a set to move in parallel, not only avoiding collision, but automatically re-aligning themselves in an emerging manner without needing to be controlled by a central command center. It is important to note that the work of [75] does employ several UAVs to carry out sound source localization; however, it does not use this information to locate and control other UAVs.
- Acoustic environment characterization: The sole presence of the UAV acoustically impacts the environment. If this impact can be measured, it can provide information about the space itself. Because of the high mobility of the UAV, the space to be characterized can be quite sizable. A noteworthy example of this is presented in [47], in which the outer walls of a building were inspected using the UAV itself as a sound source. We believe that this idea can be generalized further. The noise of the UAV could be used as a type of reference with which an acoustic space in which reverberation is a factor (such as classrooms, concert halls, auditoriums, etc.) can be characterized and diagnosed in a finer manner compared to traditional methods. It is important to mention that it may be tempting to state that this application scenario is a step away from remotely sensing ground features. However, this would require a high-resolution characterization, which typically employs a highly directed type of signal; an acoustic signal can do this, but requires a high amount of power and hardware, which is impractical to carry on a UAV.

### 7.4. Limitations

Although limitations have been described throughout this writing, they are summarized for the benefit of the reader here.

Since current popular implementations of UAVs are relatively small, the amount of space that can be dedicated for microphone mounting is quite limited. In addition, but related to this, the amount of hardware that can be mounted on-board is directly bounded by the weight the UAV is able to carry. This amounts to either requiring a moderately sized UAV to have space and be able to carry all the hardware needed for good audio perception, or developing audio-based techniques that do not require as much hardware to achieve good audio perception.

In turn, good audio perception is impacted directly by the highly dynamic acoustic environment that the UAV is usually a part of. All of the application scenarios described in the last section involve multiple sound sources that are highly mobile, with continuously changing frequency components, and with acquisition channels constantly bombarded not only with external noises (such as wind), but also with a substantial amount of ego-noise. The latter is especially significant, since it not only changes through time depending on whether the UAV is moving and how it is moving, but can be fed back rapidly multiple times depending on the reverberation characteristics of the environment (if it is close to a wall, for example).

All of this results in striking a balance between the UAV, the audio hardware, and the technique to be used. It can be argued that finding this balance is a multi-objective optimization problem, defined by a three-pronged interdependent loss function. If this is true, this problem may ultimately have several optimal solutions, each of which may be appropriate for only a few application scenarios. Thus, a one-size-fits-all combination of UAV, audio hardware, and technique may not exist.

### 7.5. Open Challenges

From the point of view of robotics, UAVs can also be seen as flying robots (aerial robotics) that could exhibit autonomous behavior. The latter is already possible when using the Global Positioning System (GPS) for autonomous flight, and extensive research has been dedicated to GPS-denied scenarios.

In the context of autonomous aerial robots, we argue that auditory perception can also contribute towards the development of intelligent autonomous robots, that is, UAVs that can fly autonomously, but that are also capable of making decisions to update their flight plans. Thus, auditory perception becomes an additional sensing mechanism that can be considered within the behavioral policy when navigating autonomously.

Therefore, we must investigate efficient ways to fuse auditory perception with another effective sensing mechanism, such as visual perception, for autonomous UAVs [86,87]. This question also involves the type of sensors to be used (regarding energy consumption and instrumentation), as well as the number of sensing units that have to be used. For instance, in [88], it is argued that the minimal set of sensors to effectively perform autonomous localization (ultimately to be used for autonomous flight) consists of a monocular camera and an inertial measurement unit (IMU). However, as we have mentioned in previous sections, auditory perception can play an essential part in applications where vision may become a handicap. Hence, we deem open for discussion the fact that more general-purpose applications involving UAVs may require one to include at least one microphone in this minimal set.

Furthermore, if auditory and visual perception (or any other type of perception) is going to be part of a UAV perception system, we must develop novel ways in which the output of these perception mechanisms can be fused, considering operation frequency, perception accuracy, and feature descriptors, among others. This may also imply intensive computational effort that could be addressed by using on-board graphical processing units (GPUs).

Deep learning via CNNs could also be exploited to process different sensor data captured by microphones, cameras, and IMUs. Hence, a current challenge is that of generating reliable datasets [46,73] that include audio, visual, inertial, and other types of sensor data with their corresponding labeling and for concrete tasks, including some initial benchmarks and metrics. This would open the door to investigating and designing CNN architectures that could extract useful features to effectively and efficiently perform auditory perception.

From the audio perception categories defined in this review, AA emerged as the category with the fewest works, and arguably the one with the most recently published works. We believe that AA is a challenging problem due to ego-noise, but also due to the on-line processing needed to perform the auditory perception within a time frame. In this sense, parallel algorithms, as well as compact CNN architectures that can run on limited-budget processors (including low-cost GPUs), are also desirable for auditory perception that can be performed on-board by micro- and nano-UAVs.

Some works have shown that ego-noise can be cancelled to a certain [89,90] extent, or it can be considered when modellling the problem [16]. However, these works can not be generalized to work for any UAV. Therefore, treatment of ego-noise is also open to research. Furthermore, there is no doubt that AA would benefit from the research done on silent or stealth UAVs [91]. Conversely, how can one deal with silent UAVs when these are the object of interest in auditory perception? Would new microphone technology be necessary? Would it be enough with software processing? These are questions that we leave open for further research.

## 8. Concluding Remarks

This review is dedicated to auditory perception in the context of UAVs. We have discussed several works that have reported the use of microphones to capture audio signals and that have some applications where UAVs are involved. To carry out our analysis and discussion, first, we proposed three categories for auditory perception: (i) air to land; (ii) land to air; (iii) air to air. This is a quick reference to indicate the origin of the sound and the location of the sensor. We have also proposed the identification of the objectives of auditory perception based on the different levels of information to be obtained from the audio source: (1) detection (if the source exists in the scene or not), (2) classification (what generated the sound), (3) localization (where the source is).

From the above, we have also presented a landscape of the number of microphones used in different works over the years. In addition, we have also discussed the software techniques reported by these works, which include deep learning via convolutional neural networks.

We have also discussed some potential applications, limitations, and open challenges. We finalize this review by stating that auditory perception for UAVs is an emerging area of research with several opportunities. In particular, we have also argued that auditory perception could complement the efforts made in the research and development of intelligent autonomous aerial robots.

## Figures and Tables

**Table 1:** Number of works with respect to the number of microphones, grouped by auditory perception category.

	# Microphones								# Works per
**Category**	**1**	**2**	**4**	**7**	**8**	**10**	**12**	**16**	**Category**
Air-to-Land	3					1			4
Land-to-Air		5	4	1	11		3	8	32
Air-to-Air		2	1		3				6
						Total of works:			42

**Table 2:** UAV types or models (if commercial) grouped by year and number of microphones. Works falling into the air-to-land category are marked with “*”.

Year	# Microphones							
	1	2	4	7	8	10	12	16
**2012**					AR drone [48]			
**2013**					AR drone [49]			
**2014**		Quad [50]	Quad [51]					Quad [52] Quad [53]
**2015**	* Phantom I [54] * Phantom II [54]							
**2016**			Quad [55] Quad pocket size [55]		Bebop [56] Bebop [57]			Quad [58]
**2017**	* Phantom 3 [59] * Phantom 4 [59] * Inspire [59] * 3DR Solo [59]			AR drone [60]	3DR IRIS [61]		Quad [24] Hexa [24] Hexa [62]	Ballon [23] Bebop [23] Pelican [23] Zion [23] Quad [63]
**2018**	* Matrice 600 pro [47]	AR drone [64] Fixed- Wing [65]	Bebop [66]		Quad [21] Quad [20] Quad [67]		Hexa [68]	
**2019**		Fixed- Wing [69] Quad [70] Quad [71]	Bebop [72]		Bebop [14] Bebop 2 [15] Matrice 100 [15] MK- Quadro [73]			
**2020**		Mavic Pro [74]	Quad [75]		Bebop 2 [16] Matrice 100 [16] Quad [76] Quad [77]	* Phantom I [78]		Quad [79] Quad [80] Hexa [81]

**Table 3:** Overview of land-to-air localization.

	Loc. Technique	Reported Performance	Noise Removal
Choi et al. [74]	CNN, energy-based features	0.065${}^{{}^{\circ}}$ error	multi-Wiener
Fernandes et al. [60]	GCC-PHAT	2.7${}^{{}^{\circ}}$ to 9.9${}^{{}^{\circ}}$ error	TLS in search
Furukawa et al. [49]	GEVD-MUSIC, Gaussian regression for noise matrix	0.7 F-value with moderate SNR	-
Hausamann [50]	Inter-aural time difference	7${}^{{}^{\circ}}$ to 23${}^{{}^{\circ}}$ error	-
Hoshiba et al. [68]	frequency-limited MUSIC	80% success, 10 dB SNR	-
Hoshiba et al. [23]	iGSVD-MUSIC	70% to 100% accuracy	-
Hoshiba et al. [24]	iGSVD-MUSIC, SEVD-MUSIC	97% success, −10 dB SNR	-
Ishiki et al. [52]	MUSIC (HARK)	-	-
Lee et al. [70]	K-medoids-based clustering using cosine distance	98% accuracy	multi-IMCRA, multi-Wiener
Manickam et al. [75]	MAP multi-UAV clustering	0.5 m to 1 m error	-
Misra et al. [64]	Intra-band beamforming	0.001 m error, 0 dB SNR	-
Nakadai et al. [62]	iGSVD-MUSIC, SEVD-MUSIC	98% success, -10 dB SNR	ORPCA
Ohata et al. [53]	iGSVD-MUSIC, correlation matrix scaling	78% correct rate, 3 m away, 2.7 m height	-
Ohata et al. [63]	iGSVD-MUSIC, correlation matrix scaling	89% correct rate, 2.7 m height	-
Okutani et al. [48]	iGEVD-MUSIC	71% correct rate	-
Salvati et al. [66]	beamforming spectral distance response	3.7${}^{{}^{\circ}}$ error, 0 dB SNR	diagonal unloading
Salvati et al. [72]	beamforming refined with face detection	>5${}^{{}^{\circ}}$ error, 0 dB SNR	diagonal unloading
Sayed et al. [51]	TDOA via cross-correlation	0.35% error	-
Sibanyoni et al. [21]	TDOA, Kalman filter, triangulation based on drone movement	80% of true value	-
Wakabayashi et al. [80]	Global nearest neighbor exploiting sound source features	0.84 m to 2.65 m of error	-
Wang et al. [20]	DOA-weighted kurtosis-histogram measures	∼1 correct ratio, 0 dB SNR	time-frequency filtering
Wang et al. [61]	TF spatial filtering, local spatial likelihood	0.9 likelihood, −10 dB SNR	-
Wang et al. [67]	TF spatial filtering, spatial confidence on non-Gaussianity, particle filtering	3.8${}^{{}^{\circ}}$ to 11.5${}^{{}^{\circ}}$ of error	-
Washizaki et al. [58]	Weighted least mean square localisation with uncertainty estimation	2.18 m of error	-
Yen et al. [77]	MVDR-based SNR response	0.0833 Haversine dist. to ground-truth	denoising autoencoder
